# Our shared global responsibility: Safeguarding crop diversity for future generations

**DOI:** 10.1073/pnas.2205768119

**Published:** 2023-03-27

**Authors:** Hannes Dempewolf, Sarada Krishnan, Luigi Guarino

**Affiliations:** ^a^Global Crop Diversity Trust, 53113 Bonn, Germany

**Keywords:** crop diversity, conservation, gene banks, plant genetic resources, food systems

## Abstract

The resilience and sustainability of food systems depend on crop diversity. It is used by breeders to produce new and better varieties, and by farmers to respond to new challenges or demands and to spread risk. However, crop diversity can only be used if it has been conserved, can be identified as the solution for a given problem, and is available. As the ways in which crop diversity is used in research and breeding change and expand, the global conservation system for crop diversity must keep pace; it must provide not only the biological materials themselves, but also the relevant information presented in a comprehensive and coherent way—all while ensuring equitable access and benefit sharing. Here we explore the evolving priorities for global efforts to safeguard and make available the diversity of the world's crops through ex situ genetic resource collections. We suggest that collections held by academic institutions and other holders that are not standard gene banks should be better integrated in global efforts and decision-making to conserve genetic resources. We conclude with key actions that we suggest should be taken to ensure that crop diversity collections of all types are able to fulfill their role to foster more diverse, equitable, resilient, and sustainable food systems globally.

The genetic diversity within and among our crops and their related cousins (crop wild relatives [CWRs]) is a vital resource for researchers, plant breeders and farmers, and its importance can only increase as climate change adds to the many challenges faced by agriculture around the world. These so-called “plant genetic resources for food and agriculture” (PGRFAs) underpin the continued productivity, nutritional value, sustainability, and resilience of agriculture. Many at the forefront of the movement to transform food systems recognize their increasing importance, from food technologists exploring sources of protein for plant-based alternatives to meat, to high-tech urban farmers hoping to supply spices old and new to trendy restaurants, to small-scale farmers in developing countries trying out new crops and farming techniques or revitalizing old and nearly forgotten ones. PGRFAs are a key example of what is widely heralded as nature-based solutions (1). Some important evidence of the impact and value of gene banks and the collections they conserve and make available was recently assembled in a special issue of the journal *Food Security* (2). This includes an economic analysis of the value of gene banks that discusses the challenges of monetary valuation of collections. One of the most compelling examples of the value of a single gene from a single gene bank accession is resistance to rice grassy stunt virus in an accession of the rice wild relative *Oryza nivara* stored at the gene bank of the International Rice Research Institute (IRRI) (3). The subsequent inclusion of the gene in several of IRRI’s most popular rice varieties helped avoid harvest losses that affected many farmers across Asia in the 1970s. Advances and significant cost reductions in genomics and phenomics over the last decade or so are fueling an increased interest by the research community in accessing and using large sets of crop diversity to develop pangenomes and similar approaches (4, 5).

However, PGRFAs are not just necessary for innovation; they are also, at the same time, a component and signifier of identity and heritage (6). Traditional crops and heirloom varieties help define culture as well as allow it to react to change. The pool of PGRFAs that has been curated by millions of farmers all around the world for millennia provides us all with myriad options to improve agriculture and food systems and adapt them to today’s and tomorrow’s challenges. It is also what makes eating an enriching cultural experience and fun.

## Why We Need Gene Banks

The use of PGRFAs by researchers, plant breeders, and farmers is of course predicated on their availability and accessibility. They cannot use what they do not have at hand. Diverse landrace and CWR populations must still exist somewhere, sufficient seeds or other propagating material from such populations must be accessible and within reach, policies and processes must be in place to make their transfer to users as frictionless as possible, and those users must know where and how to obtain what they need in the first place.

Over the past 50 y, many facilities, known as “gene banks,” have been established around the world to store PGRFAs, mainly but not exclusively in the form of seeds, and facilitate the process whereby prospective users can obtain them whether for research, plant breeding, or direct use in production (7). This has proven to be necessary because, while the long-term importance of diversity in situ in protected areas and farmers’ fields is widely recognized, many factors work against its continued survival there in the short term, and in any case, access to the places where crop diversity exists, even where these are known, is often difficult (8). It has proven to be possible because most seeds can be stored for the long term relatively easily and cheaply if kept dry and cold. Gene banks are supported by phytosanitary systems (9) to make transfers of material to users safe and by access and benefit-sharing regimes to ensure that such transfers are fair and equitable (10–13). Gene banks are an effective complement and backup to in situ and on farm conservation, and they also make access to diversity easier and safer. The importance of gene banks to food and nutritional security is recognized in Target 2.5 of the Sustainable Development Goals (SDGs; although this target is yet to be fully realized) (14).

By 2020, maintain the genetic diversity of seeds, cultivated plants and farmed and domesticated animals and their related wild species, including through soundly managed and diversified seed and plant banks at the national, regional and international levels, and promote access to and fair and equitable sharing of benefits arising from the utilization of genetic resources and associated traditional knowledge, as internationally agreed.

## Generations: The Evolution of Gene Banks

Much progress has been made in conserving PGRFAs in gene banks. The Second Report on the State of the World’s Plant Genetic Resources for Food and Agriculture suggested that there were 1,750 crop diversity collections in the world in 2009, 130 of them with >10,000 accessions (15). While it is difficult to come to a definite determination, it is likely that the number of unique accessions among these is around 2 million (15).

These gene banks come in a variety of types, each with different roles, responsibilities, strengths, and weaknesses.

1) International and regional gene banks. A number of gene banks are recognized under Article 15 of the International Treaty on Plant Genetic Resources for Food and Agriculture (ITPGRFA; Plant Treaty) as international collections. The majority are part of the CGIAR system of international agricultural research centers, and they play a central role in the global system of ex situ conservation of PGRFA by conserving global collections of major staple crops and making them available worldwide according to the highest scientific standards and norms (15–17). They are well placed to ensure the genetic integrity and long-term survival of the materials in their care through up-to-date conservation, regeneration, and safety backup processes and are able to distribute these germplasm materials safely and speedily all over the world. They are used extensively not only by breeders but also by researchers (16). Because of their crucial global importance, the Global Crop Diversity Trust was established to ensure their sustainable long-term funding, but its endowment is still not sufficiently capitalized to fully support the basic operations of all the Article 15 gene banks. In addition, a small number of regional gene banks also exist. They play a similarly important role in some geographic regions, where national governments decided to work together to establish such regional facilities to enhance cooperation on genetic resource conservation. Important examples here are the Nordic Genetic Resource Center (NordGen) in the Nordic countries and the Plant Genetic Resources Centre of the Southern African Development region (SPGRC).
2) National gene banks. Almost every country in the world has a more or less extensive network of crop diversity collections managed by local communities, universities, breeding programs, and national agricultural research institutes. Normally, a single national gene bank represents, if not coordinates, this network and has unique expertise in the crop diversity that is important at national and local levels. In some cases, such as the United States and Brazil, centralized backup facilities conserve safety duplicates of different collections spread all around the country. National gene banks work not only as repositories of local crop diversity but also as major conduits for introducing interesting new diversity from outside the country. They conserve this crop diversity but also interface with the whole range of institutions and stakeholders that require that diversity within the country, including breeding programs and farmers. National gene banks play a pivotal role in the global system in facilitating the sharing of crop diversity across borders and in scaling up the impacts of the international collections and their associated global breeding programs. Collaboration between international, regional, and national gene banksand improving the capacity of the latter—are needed to increase the levels of understanding, use, and adaptation of crop diversity to meet the challenges of climate change. With adequate resourcing, including for infrastructure upgrades and capacity building, national gene banks can play an active, indeed proactive, role in national, regional, and also international efforts to supply diversity of a full range of crops to breeding programs and farmers across the range of agroecological conditions in their countries and in sharing data and new materials back with the international gene banks to everyone’s benefit. The National Plant Germplasm System (NPGS) of the United States Department of Agriculture (USDA) is an example of a large national gene bank network that is successful in achieving many of these goals. Unfortunately, the resourcing of national gene banks is not always consistently adequate.
3) Global backup. Perhaps the world’s best-known gene bank is not strictly speaking a gene bank at all, in that it does not share its contents with others: the Svalbard Global Seed Vault located on the Svalbard archipelago in the far north of Norway, well beyond the Arctic Circle. The role of this facility in the global system of ex situ crop diversity conservation is to serve as the ultimate backup facility for other gene banks to safety duplicate their orthodox seeds. However, the world’s unique crop diversity is not yet fully backed up in the vault (18, 19) for a variety of technical, financial, and political reasons. Particularly at risk are crops that cannot easily be conserved as seeds for the long term. For such crops, normally conserved in field gene banks and in vitro, it is crucial that investments are made to develop conservation backups in cryogenic storage (20, 21).
4) Working or research collections. Other PGRFA collections that typically do not function in the way a more “conventional” gene bank does are those that have been established as part of a specific, often short-term, research project, academic initiative, or breeding program. Such collections are sometimes poorly documented, and their legal status is uncertain. As a result, they are often inaccessible to external users and also highly threatened, as academic institutions are often reluctant to take on uncertain legal liabilities when researchers are no longer able to take care of them on their own. They deserve much more recognition of their strategic importance and need funding to conserve them and make them available at least over the short to medium term. In many cases, this would be most effectively accomplished by transferring such collections to more established gene banks that have the capacity and financial resources to take on such responsibilities. This is the approach implemented by the Global Crop Diversity Trust over the past decade in its project to safeguard and use CWRs for climate change adaptation, which included supporting prebreeding researchers to deposit valuable prebred materials in major gene banks (22, 23). We say more about these collections below.
5) Community-based collections. There is a growing number of community seed banks, seed exchange networks, hobby garden clubs, etc. around the world conserving crop diversity (24). These collections are often rich in unique materials and very actively used, although perhaps by relatively few very committed individuals. With some exceptions, however, these collections are typically not managed according to long-term gene bank standards but rather in a much more dynamic way, with frequent grow outs and much exchange and less attention paid to genetic integrity and documentation. National, regional, and international gene banks could do more to support these community-based initiatives and ensure that materials that are recognized as valuable and unique are also backed up in facilities that are well equipped to conserve and research materials for the long term. Likewise, gene banks could be more active in the “rematriation” of material to communities (24, 25).

**Fig. 1.**
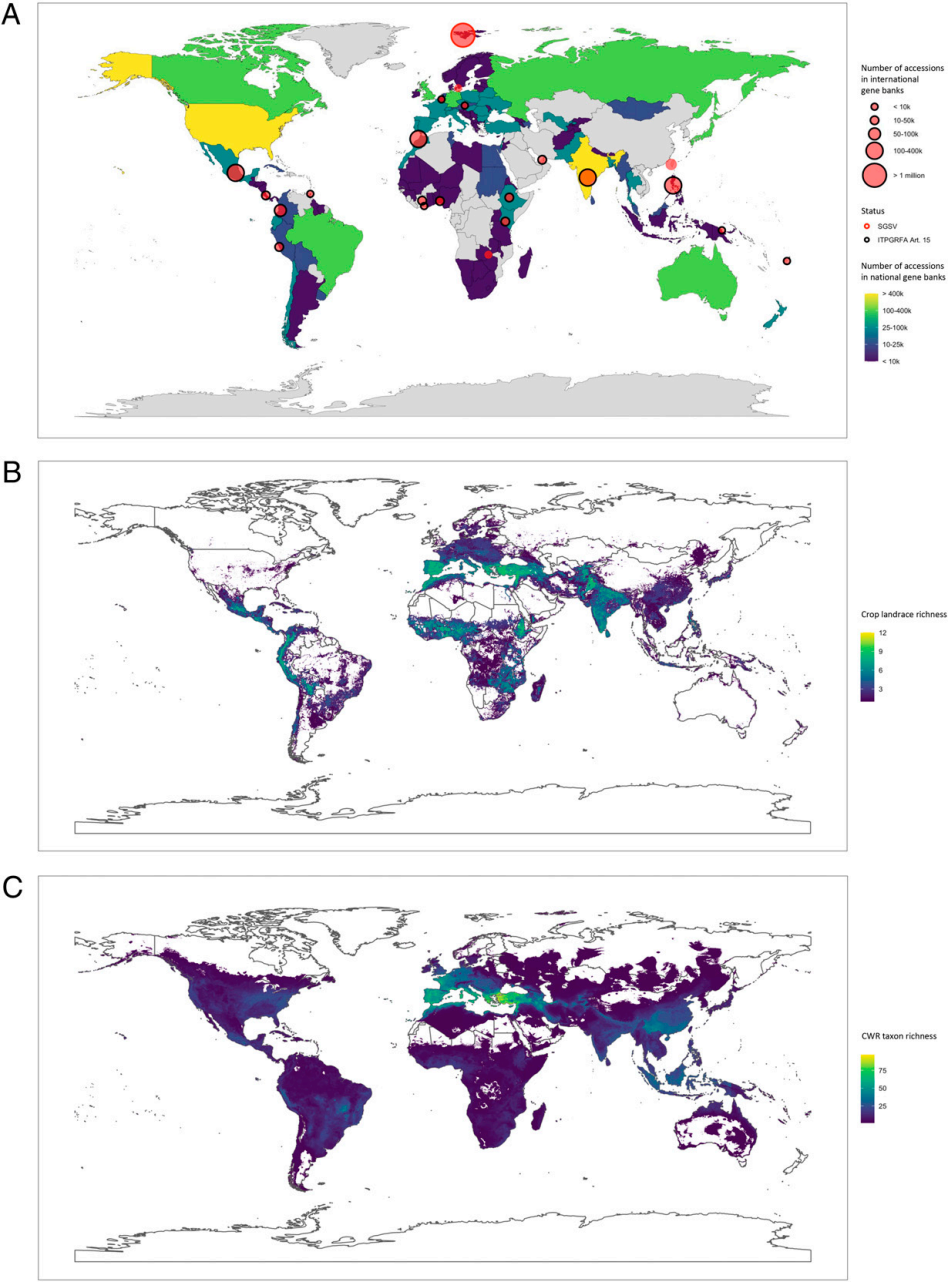
Comparison of plant genetic resource richness in ex situ conservation facilities as well as in situ in the field and in the wild. (*A*) The number of accessions in national genetic resource conservation facilities as reported to the FAO World Information and Early Warning System on Plant Genetic Resources for Food and Agriculture (WIEWS) database (https://www.fao.org/wiews/en/) is shown in different colors. The geographic locations of international, including regional, gene banks around the world are indicated by circles. The size of the circles corresponds to the reported number of PGRFA accessions in international gene banks. Information on the holdings of the Svalbard Global Seed Vault (SGSV) was retrieved from the Seed Portal (https://seedvault.nordgen.org/), and information on Article 15 status under the Plant Treaty was retrieved from the Plant Treaty website (https://www.fao.org/plant-treaty/areas-of-work/the-multilateral-system/collections/en/). (*B*) Modeled crop landrace group richness of 25 major crops. Modified from ref. 3. (*C*) Modeled richness of 1,076 CWR taxa related to 81 crops. Modified from ref. 36.

Different types of gene banks can be found in many parts of the world, often distantly located to areas of particular richness of the crop landrace diversity or CWR diversity they conserve (Fig. 1). No country in the world is self-sufficient when it comes to crop diversity, which is why it is key that international policy frameworks, such as the Plant Treaty (*The ITPGRFA*), recognize this genetic diversity as a global public good that needs to be preserved for humankind in a joint international effort for many generations to come. Coordination is needed among different gene banks (and different types of gene banks), and also between gene banks and their user communities, to make the most of limited conservation resources. This is the reason behind the Global Crop Diversity Trust’s efforts over the past two decades to bring together conservation practitioners specializing in different crops to develop global crop conservation strategies (26). Similarly, coordination of PGRFA conservation efforts can work well at a regional level, as shown by the formal European (European Cooperative Programme for Plant Genetic Resources [ECPGR]), Nordic (Nordic Genetic Resource Center [NordGen]), Pacific (Pacific Agricultural Plant Genetic Resources Network [PAPGREN]), southern African (Southern African Development Community's Plant Genetic Resource Centre [SPGRC]), and North American (Plant Genetic Resources Network for North America [NORGEN]) networks and such less-structured networking arrangement as the “Simposio de Recursos Genéticos para América Latina y el Caribe” in Latin America and the Caribbean. The most advanced of such efforts is perhaps “A European Gene Bank Integrated System (AEGIS)” project in Europe (27), which aims to identify unique accessions across European gene banks for regional-level (as opposed to only national-level) management and, possibly, funding. However, the necessary financial support to sustain such regional collaboration and coordination is often inadequate, which means that several PGRFA networks that were formerly more active are currently dormant or have been discontinued.

The flow of germplasm among gene banks and between gene banks and their users is considerable, thanks in no small measure to international agreements that provide a solid legal basis for access and benefit sharing, first and foremost the Multilateral System for Access and Benefit Sharing (MLS) established under the Plant Treaty (28–31). In 2021 alone, the international gene banks of the CGIAR distributed 96,590 seed samples of 63,788 accessions to 91 countries around the world, with 51% of these external distributions destined for universities or research institutions (32).

Access to PGRFAs and the equitable sharing of benefits derived from their use are at the heart of the MLS. It is not a perfect system, and its flaws are widely recognized. However, improvements are under discussion among policy makers to ensure that it continues to evolve to better meet the evolving expectations of all stakeholders with regard to both biological materials and the data that are connected to them.

## The Plant Treaty

The ITPGRFA is an international agreement that supports the conservation and sustainable use of PGRFAs as well as the fair and equitable sharing of benefits arising from the use of PGRFAs. Its Secretariat is hosted by the Food and Agriculture Organization of the United Nations (FAO) in Rome. At the core of the ITPGRFA is the MLS, which provides a legal basis for gene banks to share materials for research, training, breeding, and conservation with each other and with users. In 2018, the MLS amounted to some 2.5 million accessions. Products derived from the use of MLS material in breeding may be commercialized, but any monetary benefits derived from this must be shared back with all participants in the system according to the agreed rules. Some 148 countries and the European Union are contracting parties. By January 2022, 6.1 million germplasm samples from the MLS had been received by users.

The effectiveness with which gene banks provide users with the material they need does depend crucially on access to data. Increasing investments over the last two decades in information systems to manage and make available data on genetic resources contained in collections (29–31, 33) are starting to pay off. The European regional data portal (EURISCO) and a growing number of national and international gene banks all share accession-level data on the global portal for genetic resource information, Genesys (https://www.genesys-pgr.org/). This now includes passport information on more than 4 million accessions. Genesys is a key component of the Plant Treaty’s Global Information System, which includes a means for providing digital object identifiers for gene bank accessions, a major advance in the documentation of PGRFAs.

Such information infrastructure is not only key for making the diversity in collections accessible and searchable, but it is also necessary for monitoring progress toward global PGRFA conservation goals. Most notable in this context is the continuing work of the FAO Commission on Genetic Resource for Food and Agriculture on the State of the World reporting and associated Global Plan of Action frameworks (15, 28). Higher-level policy frameworks, such as the Aichi Biodiversity Targets as well as the SDG targets, are also important in the process of resource allocation by national governments and others in support of conservation efforts. Although the Aichi Biodiversity targets and the associated SDG Target 2.5 expired in 2020, a new set of global targets is under negotiation in the form of the post-2020 Global Biodiversity Framework under the Convention on Biological Diversity.

## Into Darkness: Important PGRFA Collections beyond Gene Banks

As alluded to above, a considerable wealth of PGRFAs is held by academic institutions and research laboratories as “temporary,” “working,” or “research” collections and also, as decommissioned breeding collections by private industry. Although reliable and comprehensive data on these are difficult to come by, the anecdotal evidence is fairly clear that they may be very significant. Often poorly documented and not available for distribution, they could be considered to be the “dark” part of the global system of ex situ conservation. Some such collections have grown organically and sporadically over the perhaps quite lengthy tenure of a single faculty member. Another example is when a laboratory develops new material, such as by single-seed descent, to purify genetically heterogeneous populations prior to genotyping. What happens to the seeds once the faculty member retires or the experiment is finished?

The unfortunate fact is that they are at great risk of being lost or destroyed, as when faculty members retire or move on or funded projects end. Academic institutions are generally poorly positioned to care for and distribute even the most valuable material for the long term. The same is true for breeding collections developed by private industry once a company decides to discontinue certain breeding programs or divest from entire crops. At best, they are handed over to more conventional gene banks that may not have the capacity or necessary funding to take on large quantities of new accessions ad hoc. At worst, they are simply abandoned.

Yet, we know that the size and uniqueness of some of these collections can be substantial. When they are made accessible (for example, by being absorbed by national or international gene banks), they are often in much demand, as they are closer to the type of materials breeders and farmers can use directly. International gene banks that have integrated genetic stocks into their collections have reported a significant increase in requests for this type of material in recent years (34).

These dark collections must come into the light. The academic community must consider the possible conservation value of the PGRFA collections they host and give specific attention to their genetic and phenotypic uniqueness as well as their possible broader value for use beyond the often narrow objectives of particular research groups or projects. On their side, publishers and research funders should require researchers to submit a plan for the sustainable management of genetic materials beyond a research project’s lifetime. Funding for this could be explicitly included in research project proposals, or an agreement could be prenegotiated with established gene banks to take on these responsibilities. Often, these materials will not need to be managed with very long-term conservation objectives, but they can be retired or decommissioned after a given period of time has elapsed and the wider community has had a chance to gauge their usefulness. However, the necessary modalities and financing for the conservation and distribution of these materials need to be agreed and secured beforehand.

In addition to academic institutions and research collections driven by specific research projects, there are hobbyists with a passion for specific plants that hold valuable PGRFA collections (35, 36) as well as collections held by botanic gardens. Some 3,000 botanic gardens conserve over 120,000 plant species worldwide, maintained ex situ as living collections and in seed banks that also include crop diversity, in particular CWRs (37, 38). There are many examples of specific botanic gardens that engage in both in situ and ex situ conservation of locally relevant PGRFAs in collaboration with local communities (39). Botanic gardens complement collections held by international, regional, and national crop gene banks and should be tapped into by users of PGRFA, encouraging mutually beneficial collaboration between agricultural research organizations and the wider plant conservation community.

## Beyond: The Future of PGRFA Conservation

Considerable progress has been made to date toward conserving PGRFAs in ex situ collections (40). However, the global climate crisis (41); rapidly changing technologies, especially in data science; and changing user demands all present challenges for today’s gene banks. Only if gene banks are able to continue to adapt will they be able to provide the crop diversity that, in turn, is so crucial for the world’s food systems to adapt. We think there are four areas where we need more and faster progress:

1) saving dark collections,
2) preempting genetic erosion and strengthening the ex situ/in situ conservation continuum,
3) adopting orphan crops, and
4) making the most of data.

We have already discussed dark collections above. We turn to the others below.

Crop genetic diversity is difficult (although becoming easier) to measure, and its value is not always adequately recognized, let alone routinely monitored. The adoption by farmers of new varieties, new crops, and new farming practices often leads to higher yields and other beneficial effects, yet it may also contribute to significant collateral damage (i.e., the loss of genetic diversity in their fields). This process of losing crop genetic diversity is called genetic erosion (8). It happens on two levels. First, numerous varied local varieties (landraces) may be replaced by a much smaller number of new modern ones in a given area—perhaps by just one. Second, these modern varieties are by definition genetically uniform compared with traditional landraces, although they may be quite genetically distinct from each other and indeed, from the landraces they are replacing.

In addition, populations of the wild relatives of crops are also often put at risk by the forces that threaten wild plants more generally—changes in land use, pollution, overharvesting, and invasive species—sometimes to the extent of risking the extinction of entire species. Nevertheless, CWRs are seldom given special status within overall biodiversity conservation planning and prioritization, despite their increasing use and importance in crop improvement and agricultural development (42).

Genetic erosion limits the future options of both plant breeders and farmers. When it—or better, the threat of it—is detected, urgent steps need to be taken to counter it, crucially including preemptive collecting and conservation in gene banks of the diversity deemed at risk. We know that gaps remain in the coverage of ex situ collections of many crops with regard to both landraces and wild relatives. Diversity threatened in the field may not be in gene banks (7, 43). Likewise, in situ conservation efforts in areas where crop diversity is still abundant need to be better supported, and ex situ and in situ conservation efforts need to work better together as part of an overall ex situ/in situ conservation continuum.

Unfortunately, there is no system in place today at the local or national level, let alone regionally or globally, to gauge the threats to crop diversity posed by different proposed or ongoing activities and processes, to monitor these threats through time, or to guide emergency remedial actions as necessary. We believe that such a system should be established as a matter of urgency.

A “real-time” monitoring system for genetic erosion could be composed of three elements: 1) a “big data” tool that employs a geographically explicit approach to automatically mine, synthesize, and analyze disparate data sources to identify likely hot spots of genetic erosion; 2) a “crowdsourcing” reporting tool (ideally a smartphone app) that allows users (e.g., farmers, extension workers, conservationists, Non-Governmental Organizations (NGOs), national focal points, protected area managers) to flag incidences of suspected threat to or loss of crop diversity in the field; and 3) a small task force of experts that carries out a preliminary assessment of perceived threats and if necessary, recommends appropriate action—with regard to both in situ and ex situ conservation activities.

Ideally, when the threat of genetic erosion is identified, resources would then be quickly mobilized from an emergency fund to implement an effective response. This will usually take the form of an emergency collecting expedition to the affected area coordinated by the national PGRFA conservation program, but it could also take longer-term forms. For example, recommendations could be made for in situ conservation interventions, including the reintroduction and restoration of crop diversity from other localities or gene banks. Such an emergency fund could be modeled after the Emergency Reserve for Gene Banks that was recently launched by the Plant Treaty and the Global Crop Diversity Trust to counteract the threat of genetic erosion in gene banks (44).

When all this is in place and functioning, the world would for the first time have a system that provides early warnings of threats to vital, unsecured crop genetic diversity and be able to act with the necessary urgency. It would also allow researchers to determine if and when the metrics of diversity increase or decline in fields or in the wild at local, regional, and global scales. This system would be an important way to guide actions and monitor progress toward the implementation of global development and conservation goals (i.e., SDG Target 2.5, Aichi Target 13, and the Global Plan of Action on PGRFAs). More importantly, it would be an enormous benefit to food system resilience worldwide.

Crucially, in monitoring genetic erosion and other conservation efforts, more attention needs to be paid to crops and useful wild species that are locally or regionally significant for human consumption but are perhaps not (yet) of wider importance (45). These species have been described as “neglected and underutilized species,” “orphan crops,” or “development opportunity crops,” and almost all of them have two things in common. 1) They are of potential importance for efforts to diversify agriculture and human nutrition on local, regional, and sometimes, even global scales (46). 2) They are inadequately researched and conserved (15, 26). If global efforts to diversify food systems are to gain pace, orphan crops must be “adopted” by gene banks and plant breeders.

Global crises are driving novel industries, new types of farming, and innovative research. These challenge gene banks to change and adapt in order to stay current and relevant. However, what is unlikely to change is the demand for disease-free, well-documented genetic materials. Accessions without data that are presented in a simple and understandable way are less likely to be used, and it is use that makes the case for conservation. Gene bank information systems must grow, expand, and become easier to use. More and better data have to be collected on an ever-increasing set of accessions, traits, and characteristics. Whether this is done by phenotyping robots in research fields or through participatory evaluation efforts with farmers (47) and whether the data are analyzed with the help of artificial intelligence (AI) or with pen and calculator, data have to be well documented, clearly linked to individual accessions, and easily available for search and download (31, 48).

Most of this evaluation of crop diversity will not be done by gene banks themselves, as the necessary expertise in all the ways of measuring all the possible different traits that may be of interest now and in the future will naturally reside mainly with the different communities of users who are driven by particular research questions, breeding goals, production, or consumption constraints. However, gene banks must have access to the resulting data if they are to be in a position to better guide future use. Much remains to be done in this area; efforts, like the ITPGRFA's Global Information System (GLIS), are certainly helpful. What is even more crucial is that the role of gene banks as data hubs is fully recognized, and gene banks, in turn, must understand and embrace how the core of their business is no longer just the maintenance and curation of biological material, but also includes facilitating access to the data that comes with the materialeven the data they do not generate themselves.

Digital data on collections, whether genotypic or phenotypic, can now be gathered much more readily through high-throughput approaches and can serve both as an essential organizing principle for collections and to rationalize their curation. An ambitious initiative would be the development of the digital infrastructure to support a worldwide visual compendium of crop biodiversity, available as part of a global digital commons. Such a compendium could form the basis for efforts to train image analysis algorithms and AI systems for a wide range of applications, including monitoring and tracking changes in crop biodiversity, training climate change prediction models to suggest best-bet germplasm material, and ultimately, providing decision-makers with a trusted data source to build more diverse and resilient food systems and landscapes.

A considerable obstacle to major advances is certainly a lack of funding for such data generation and curation, as most project funding is focused on the relatively short-term use of materials for particular research questions or for breeding efforts in which impacts are measured much farther downstream, in particular in the improvement of farmers’ incomes and livelihoods. Few funders recognize that to reap the benefits of agrobiodiversity, much more has to be invested in the conservation, evaluation, and data management of collections. Without systematic and large-scale investments in fundamental gene bank infrastructure and operations to enable an effective two-way flow of information and germplasm between gene banks and users (49), the potential of crop diversity to increase food system sustainability and resilience will not be realized.

Saving dark collections, monitoring and preempting genetic erosion, taking on orphan crops, and managing data better are all big additional tasks for gene banks that are often already struggling. However, the world’s gene banks are many, and their staff members are experienced, capable, and committed. There is much diversity among gene banks and their staff, and that is a great strength that must be fostered and embraced. If they collaborate and share responsibilities, if they truly serve their users, and crucially, if they are properly resourced, gene banks will live long and prosperas will the PGRFAs in their care, the farmers on which they depend, and all of us who depend on farmers.

## Data Availability

There are no data underlying this work.
